# Neuroprotective Effects of Herbal Formula Yookgong-Dan on Oxidative Stress-Induced Tau Hyperphosphorylation in Rat Primary Hippocampal Neurons

**DOI:** 10.3390/biology15030294

**Published:** 2026-02-06

**Authors:** Hyunseong Kim, Jin Young Hong, Changhwan Yeo, Hyun Kim, Wan-Jin Jeon, Junseon Lee, Yoon Jae Lee, In-Hyuk Ha

**Affiliations:** Jaseng Spine and Joint Research Institute, Jaseng Medical Foundation, Seoul 135-896, Republic of Korea; biology@jaseng.org (H.K.); vrt23@jaseng.org (J.Y.H.); duelf2@jaseng.org (C.Y.); khyeon94@jaseng.org (H.K.); cool2305@jaseng.org (W.-J.J.); excikind@jaseng.org (J.L.); goodsmile@jaseng.org (Y.J.L.)

**Keywords:** hippocampus, Yookgong-dan, oxidative stress, Alzheimer’s disease, glycogen synthase kinase 3β

## Abstract

This study sought to evaluate the neuroprotective effects of YGD in an oxidative stress-induced Alzheimer’s disease (AD)-like cellular model and to elucidate the underlying molecular pathways, with a focus on tau phosphorylation, Aβ accumulation, and antioxidant defense mechanisms. Rat primary hippocampal neurons were exposed to hydrogen peroxide to induce oxidative stress. The effects of YGD on neuronal viability, neurite outgrowth, and synaptic integrity were assessed using the immunodetection of microtubule-associated protein 2 (MAP2), postsynaptic density protein 95 (PSD-95), and synapsin-1. Levels of phosphorylated tau and Aβ were quantified, and the involvement of extracellular signal-regulated kinase (ERK), glycogen synthase kinase 3β (GSK3β), and nuclear factor-erythroid 2-related factor-2 (Nrf2) pathways was examined. Additionally, in silico molecular docking studies targeting the ATP-binding site of GSK3β were conducted to screen major phytochemicals from the ten medicinal herbs constituting YGD. YGD markedly enhanced neuronal viability under oxidative stress, promoted neurite extension, and increased synaptic marker expression (MAP2, PSD-95, and synapsin-1). Treatment reduced phosphorylated tau by suppressing ERK and GSK3β activation and significantly decreased Aβ accumulation. YGD also upregulated antioxidant defenses via the activation of the Nrf2 pathway. Docking simulations identified oleanolic acid (from *Cornus officinalis*) as the most potent GSK3β binder (−9.86 ± 0.40 kcal/mol), forming stable interactions with ARG96, ASN95, and GLU97. Additional compounds, including alisol C, drypemolundein B, and friedelin, demonstrated favorable binding energies and engaged key ATP-binding site residues. YGD confers neuroprotection through the integrated modulation of tau phosphorylation, Aβ pathology, and oxidative stress, partly via the multi-target engagement of GSK3β by its constituent phytochemicals. These findings support that YGD attenuates oxidative stress-induced AD-like cellular alterations.

## Simple Summary

Oxidative stress contributes to neuronal damage and is linked to Alzheimer’s disease-related pathological changes, including tau hyperphosphorylation and amyloid-β (Aβ) accumulation. Although Yookgong-dan (YGD) offers benefits for cognitive enhancement and stress reduction, its specific mechanisms of action in the context of Alzheimer’s disease remain unexplored. Here, we examined the effects of YGD, a traditional herbal formula, in rat primary hippocampal neurons exposed to hydrogen peroxide as an oxidative stress-induced cellular model. YGD improved neuronal viability, promoted neurite outgrowth, and increased synaptic marker expression under oxidative stress conditions. Additionally, YGD reduced phosphorylated tau and Aβ levels, accompanied by the modulation of extracellular signal-regulated kinase and glycogen synthase kinase 3β signaling and activation of the nuclear factor-erythroid 2-related factor-2-associated antioxidant response. These findings suggest that YGD modulates oxidative stress-associated neuronal alterations relevant to Alzheimer’s disease-related pathology, although further in vivo validation is required.

## 1. Introduction

Hippocampal neurons are critical for learning and memory formation. As part of the limbic system, the hippocampus is essential for spatial memory and converting short-term memory into long-term memory [1,2,3]. Neuronal damage and the progression of neurodegenerative diseases, such as Alzheimer’s disease (AD), are strongly influenced by oxidative stress. AD, the most common neurodegenerative disorder, is characterized by progressive cognitive decline, memory loss, and behavioral changes [4,5,6]. On a molecular level, AD is marked by the abnormal accumulation of amyloid-β (Aβ) plaques and neurofibrillary tangles (NFTs) composed of hyperphosphorylated tau protein. These pathological features contribute to synaptic dysfunction, neuronal loss, and ultimately, brain atrophy [7,8]. Among the kinases implicated in AD pathology, glycogen synthase kinase 3β (GSK3β) plays a central role. GSK3β is a critical enzyme in AD pathology, known to drive tau hyperphosphorylation, Aβ accumulation, and neuroinflammatory responses. Dysregulation of GSK3β activity has been consistently associated with synaptic dysfunction, neuronal loss, and cognitive decline in both experimental models and patients with AD [9].

Oxidative stress is a key driver of AD progression, exacerbating Aβ accumulation and tau hyperphosphorylation, both of which accelerate neurodegeneration [7,8,10,11]. This pathological condition arises from an imbalance between the production of reactive oxygen species (ROS) and the antioxidant defenses of the body, leading to cellular damage [12,13]. Hydrogen peroxide (H_2_O_2_), a widely studied ROS, is commonly used to induce oxidative stress in in vitro models of neurodegeneration [14]. H_2_O_2_ exposure increases tau phosphorylation through the activation of kinases, such as glycogen synthase GSK3β and extracellular signal-regulated kinase (ERK), which are closely linked to AD progression. Targeting these kinases and enhancing antioxidant responses represent promising strategies for mitigating AD-related neurodegenerative changes [15,16,17].

Yookgong-dan (YGD) is a traditional herbal formulation that combines two well-established remedies in East Asian medicine, Gongjin-dan (GJD) and Yukmijihwang (YMJ) [18]. Previous studies have reported that YGD exhibits diverse biological activities, including immune-enhancing effects, anti-fatigue properties, and neuroprotective potential. YGD has been shown to ameliorate cyclophosphamide-induced immunosuppression and to modulate immune-related signaling pathways in animal models. Additionally, related formulations have been reported to improve cognitive function and resistance to stress [18,19]. Notably, a previous study has reported that the herbal prescription Youkongdan (an earlier formulation of YGD) improved hippocampus-dependent memory performance and reduced neuronal cell loss in the CA1 region of the hippocampus in rodent models of cerebral ischemia and scopolamine-induced impairment, indicating potential neuroprotective and cognitive benefits associated with YGD treatment [19]. GJD, renowned for its antifatigue, anti-amnesic, and antiaging properties, is a blend of herbs with proven antioxidant, anti-inflammatory, neuroprotective, and immune-enhancing effects [20,21,22,23,24]. Similarly, YMJ is composed of six medicinal plants traditionally used to treat kidney disorders, diabetes, neurological conditions, and osteoporosis, and it has been shown to improve memory, boost immune function, and support recovery from physical weakness [25,26,27]. Although YGD is believed to leverage the synergistic effects of GJD and YMJ, offering benefits for cognitive enhancement and stress reduction, its specific mechanisms of action in the context of AD remain unexplored.

We hypothesized that YGD attenuates oxidative stress-induced AD-like neuronal alterations by modulating GSK3β–tau signaling through antioxidant mechanisms, including the activation of the Nrf2 pathway.

## 2. Materials and Methods

### 2.1. Preparation of YGD

YGD was formulated using a blend of ten medicinal herbs: *Rehmannia glutinosa* (Gaertn.) Steud. (260 g per 1 kg), *Angelicae radix* (130 g), *Dioscorea polystachya* Turcz. (130 g), *Cornus officinalis* Siebold & Zucc. (130 g), *Cervi pantotrichum* (65 g), *Wolfiporia extensa* (Peck) Ginns (65 g), *Alisma canaliculatum* A.Braun & C.D.Bouché (65 g), *Moutan cortex radices* (65 g), *Musk* (65 g), and *Aquilaria agallocha* Roxburgh (25 g). All medicinal materials were purchased from Green Myeongpoom Pharmaceutical Co. (Namyangju-si, Gyeonggi-do, Republic of Korea). The preparation procedure of YGD powder was consistent with that described in a previously published study [18]. The mixture was dried for 24 h at 70 °C, ground into a fine powder, and stored at −20 °C until use. For experiments, the YGD powder was dissolved in phosphate-buffered saline (PBS; Gibco, Waltham, MA, USA) at a concentration of 10 mg/mL, then filter-sterilized using a 0.45 μm syringe filter (Advance Co., Ltd., Tokyo, Japan) to ensure sterility.

### 2.2. Primary Culture of Rat Hippocampal Neurons

Hippocampal neurons were isolated from postnatal day 1–2 male SD rat pups (Samtako Bio, Gyeonggi-do, Republic of Korea) under anesthesia induced by hypothermia and isoflurane (BK Pharm, Goyang-si, Republic of Korea). Following decapitation, the brains were carefully dissected to extract the hippocampi, after which the meninges were gently removed with fine forceps, minimizing damage to surrounding tissues. The isolated hippocampi were collected in cold Dulbecco’s PBS (DPBS) and subjected to enzymatic dissociation using papain and DNase I (Worthington Biochemical, Lakewood, NJ, USA). The enzymatic activity was neutralized using an ovomucoid protease inhibitor and bovine serum albumin to minimize tissue degradation. The cells were gently triturated to ensure complete dissociation, followed by centrifugation. The resulting cell pellet was resuspended in Neurobasal Plus medium (Gibco) supplemented with 1% penicillin/streptomycin (Thermo Fisher Scientific, Waltham, MA, USA), 1× GlutaMax (Thermo Fisher Scientific), and 1× B27 (Gibco). The dissociated neurons were seeded in various formats: 1 × 10^6^ cells per well in 6-well plates, 3 × 10^4^ cells per well in 96-well plates, or 2 × 10^5^ cells per well on 12 mm circular coverslips placed in 24-well plates. All culture surfaces were pre-coated with 20 μg/mL poly-D-lysine and 10 μg/mL laminin (Gibco-BRL).

### 2.3. Experimental Timeline and Workflow

The neuroprotective effects of YGD were evaluated in hippocampal neurons exposed to 200 μM H_2_O_2_ and varying concentrations of YGD (10, 25, and 50 μg/mL) through three distinct experimental protocols. For neurite outgrowth assessment, neurons were cultured for 1 day before co-treatment with YGD and H_2_O_2_ for 24 h. Then, neurite growth was analyzed 24 h after treatment. For synapse formation and marker analysis (including Nrf2 and Aβ 1–42), neurons were allowed to mature in culture for 14 days before co-treatment with YGD and H_2_O_2_ for 24 h. Samples were collected on the following day and processed for immunocytochemical analysis. For protein expression studies using Western blot analysis, neurons were cultured in six-well plates for 14 days and co-treated with YGD and H_2_O_2_ for 30 min before samples were collected for ERK, GSK3β, and tau protein analyses.

### 2.4. Cell Counting Kit-8 (CCK-8) Assay

Cell viability was evaluated using the CCK-8 assay (Dojindo, Kumamoto, Japan) following co-treatment of neurons with YGD and H_2_O_2_ for 24 h. Neurons cultured in 96-well plates were analyzed after 1 or 14 days of growth. On day 3 or 15, 10% CCK-8 solution was added to each well and then incubated for 4 h. Absorbance was measured with an Epoch microplate reader (BioTek, Winooski, VT, USA) to quantify cell viability, which was expressed as a percentage relative to the control cells.

### 2.5. Live/Dead Cell Assay

To further assess cell viability, a Live/Dead Cell Viability Kit (Molecular Probes, Eugene, OR, USA) was used. Hippocampal neurons cultured on 12 mm circular coverslips were treated with 2 μM calcein-AM and 4 μM ethidium homodimer-1 in DPBS for 15 min at 37 °C. Following treatment, the coverslips were washed with DPBS and mounted using mounting medium (Dako Cytomation, Carpinteria, CA, USA). Images of the live (green fluorescence) and dead (red fluorescence) cells were captured using a confocal microscope (Eclipse C2 Plus; Nikon, Tokyo, Japan) at 100× magnification from six randomly selected fields per sample. Fluorescence intensity was quantified using ImageJ software (version 1.37, National Institutes of Health, Bethesda, MD, USA; *n* = 10), and the live/dead ratio was calculated.

### 2.6. Immunocytochemistry

Cells were fixed with 4% paraformaldehyde for 30 min, washed three times with PBS (5 min each), permeabilized using 0.2% Triton X-100 in PBS for 5 min, and washed twice with PBS. To block non-specific binding, cells were incubated with 2% normal goat serum in PBS for 1 h at 25 ± 1 °C. The samples were incubated overnight at 4 °C with primary antibodies diluted in 2% normal goat serum, including anti-microtubule-associated protein 2 (MAP2; guinea pig polyclonal, 1:1000, Synaptic Systems, Göttingen, Germany), anti-MAP2 (rabbit polyclonal, 1:1000, Abcam, Cambridge, UK), anti-Aβ (1:200, Bioss, Woburn, MA, USA), anti-Nrf2 (1:200, Abcam), anti-synapsin-1 (1:500, Synaptic Systems), and anti- postsynaptic density protein 95 (PSD-95; 1:200, Millipore, Sigma, Burlington, MA, USA). After washing three times with PBS (5 min each), the cells were incubated with fluorescently conjugated secondary antibodies (1:300, Jackson ImmunoResearch Labs, West Grove, PA, USA) for 2 h at 25 ± 1 °C. Finally, cells were washed, stained with 4′,6-diamidino-2-phenylindole (Tokyo Chemical Industry Co., Ltd., Tokyo, Japan) for 10 min at 25 ± 1 °C, washed again, mounted in a fluorescence mounting medium (Dako Cytomation), and imaged using a confocal microscope (Eclipse C2 Plus, Nikon). For neurite outgrowth analysis, seven representative images were captured at 200× magnification using fixed acquisition settings. Neurite outgrowth was measured using ImageJ software (version 1.37).

### 2.7. Western Blot

For Western blot analysis, hippocampal neurons were homogenized in radioimmunoprecipitation assay buffer containing protease and phosphatase inhibitors (Millipore) for 30 min [27]. The protein lysates were separated using 8% sodium dodecyl sulfate–polyacrylamide gel electrophoresis and transferred to polyvinylidene difluoride membranes (Millipore) via electroblotting at 100 V for 90 min. Membranes were blocked with 5% non-fat skim milk (BD Biosciences, San Jose, CA, USA) for 1 h at 25 ± 1 °C before incubation with primary antibodies (Table 1) overnight at 4 °C. After washing, the membranes were incubated with the corresponding horseradish peroxidase-conjugated secondary antibodies for 2 h at 25 ± 1 °C. Protein bands were visualized using enhanced chemiluminescence reagents (Bio-Rad, Hercules, CA, USA) and detected using an Amersham Imager 600 (GE Healthcare Life Sciences, Uppsala, Sweden). ImageJ software was used for band quantification.

### 2.8. In Silico Molecular Docking Analysis

To evaluate the potential interactions between phytochemicals derived from YGD and GSK3β, molecular docking analysis was conducted using AutoDock Vina (version 1.1.2). The three-dimensional (3D) structure of human GSK3β was obtained from the AlphaFold Protein Structure Database (UniProt ID: P49841; https://alphafold.ebi.ac.uk/). The AlphaFold-predicted structure was selected instead of an experimentally solved crystal structure because most GSK3β structures available in the Protein Data Bank represent holo-form conformations complexed with specific inhibitors, which may introduce ligand-induced conformational bias. In contrast, the AlphaFold model provides a ligand-independent, apo-like structure, allowing for a more objective evaluation of binding interactions for diverse phytochemicals during the docking-based screening stage. All phytochemicals derived from the medicinal herbs comprising YGD with reported and available three-dimensional structural information were included in the initial docking screening. Phytochemical information was collected from the previously published literature and public chemical databases. For each constituent herb of YGD, all known compounds with available 3D structures were subjected to molecular docking analysis without prior filtering based on biological activity or chemical class. Using this comprehensive screening strategy, approximately 1900 phytochemicals were docked against GSK3β. From this large-scale docking analysis, the ten compounds exhibiting the strongest predicted binding affinities were selected for subsequent analysis and visualization. Protein preparation, including the removal of water molecules and the addition of polar hydrogens, was performed using AutoDock Tools (version 1.5.7). Phytochemical constituents were comprehensively collected from the previous literature and phytochemical databases, including PubChem (https://pubchem.ncbi.nlm.nih.gov/) and the Korean Traditional Knowledge Portal (https://www.koreantk.com, accessed on 27 August 2025). For most of the ten medicinal herbs comprising YGD, representative bioactive compounds were retrieved from these databases. However, for *Alisma canaliculatum Braun & Bouché*, *Moutan cortex radices*, and *Musk*, relevant phytochemical data were not available in the phytochemical databases. Therefore, for these three herbs, compound selection was based on sources from the literature that reported major or pharmacologically relevant constituents [28,29,30,31]. All compounds were downloaded in SDF format and converted into PDBQT format for docking. Docking simulations were conducted by targeting the ATP-binding site of GSK3β, and binding affinities were recorded in kcal/mol. For molecular docking, a grid box was defined to encompass the ATP-binding site of GSK3β. The grid box center was set at X = 8.67, Y = −6.533, and Z = −6.693, with dimensions of 26.8 × 21.6 × 18.2 Å. The energy range parameter was set to 4 kcal/mol to allow for the identification of diverse binding poses during the screening stage. The exhaustiveness value was set to 16, which is higher than the default setting, to ensure a more thorough and precise exploration of the conformational space. The docking poses were ranked on the basis of binding energy scores. Protein–ligand interactions were visualized and analyzed using PyMOL (version 2.5) and Discovery Studio Visualizer (version 24), focusing on hydrogen bonding, hydrophobic contacts, and π–π interactions relevant to GSK3β inhibition.

### 2.9. Statistical Analysis

Data are expressed as the mean ± standard deviation. Statistical analysis was performed using one-way analysis of variance, followed by Tukey’s post hoc test, using GraphPad Prism (GraphPad Software version 8, San Diego, CA, USA). Significance levels were set at # *p* < 0.05, ## *p* < 0.01, and #### *p* < 0.0001 versus the blank group; and * *p* < 0.05, ** *p* < 0.01, *** *p* < 0.001 and **** *p* < 0.001 versus the H_2_O_2_ group.

## 3. Results

### 3.1. YGD Promotes Cell Survival in H_2_O_2_-Stressed Hippocampal Neurons

**Figure 1:**
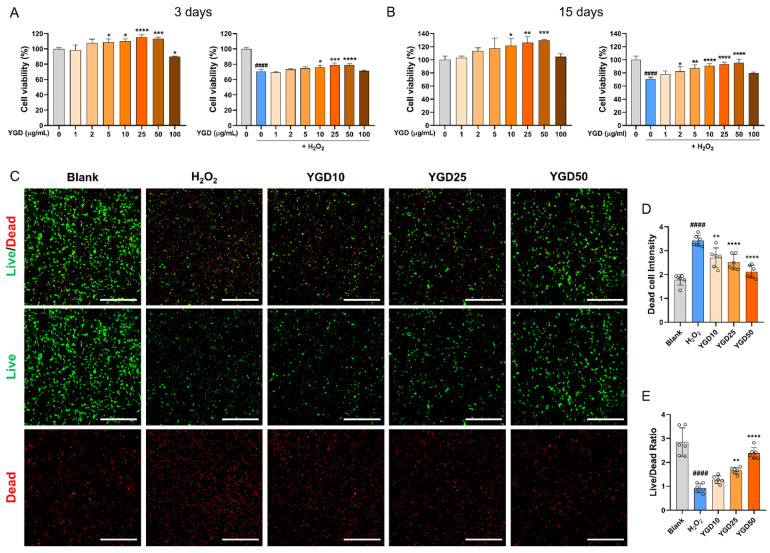
YGD enhances cell viability and provides neuroprotection to hippocampal neurons under H_2_O_2_-induced oxidative stress. (**A**) CCK assay results following the treatment of hippocampal neurons, cultured for 2 days, with YGD at concentrations of 1–100 μg/mL, either alone or in combination with H_2_O_2_, and analyzed on day 3. (**B**) CCK assay results for hippocampal neurons cultured for 14 days, treated with YGD at concentrations of 1–100 μg/mL, either alone or in combination with H_2_O_2_, and analyzed on day 15. (**C**) Representative images from the live/dead assay on day 3, where hippocampal neurons were cultured for 2 days and treated with optimal concentrations of YGD (10, 25, and 50 μg/mL) alongside the H_2_O_2_ and blank groups. The white scale bar represents 400 μm. (**D**) Quantitative analysis of the live/dead assay showing the dead cell intensity. (**E**) Quantitative analysis of the live/dead assay displaying the live/dead ratio. Data are presented as the mean ± standard deviation. Significant variations were evaluated using one-way analysis of variance (ANOVA) coupled with Tukey’s post hoc analysis. Significance levels are denoted as follows: #### *p* < 0.0001 compared with the blank group; * *p* < 0.05, ** *p* < 0.01, *** *p* < 0.001, and **** *p* <0.0001 compared with the H_2_O_2_ group.

To determine the optimal dose of YGD for enhancing cell viability and its neuroprotective effects under H_2_O_2_-induced oxidative stress, a CCK assay was conducted. Hippocampal neurons cultured for 2 and 14 days were treated with varying YGD concentrations (1–100 μg/mL). The assay was performed both with YGD alone and in combination with H_2_O_2_. On day 3, YGD alone significantly increased cell viability compared with the untreated group, starting at 5 μg/mL, with this effect persisting up to 50 μg/mL. However, at 100 μg/mL, YGD caused a significant reduction in cell viability, suggesting potential cytotoxicity at this dose. Under co-treatment with H_2_O_2_, YGD at 10–50 μg/mL significantly improved cell viability compared with the H_2_O_2_-only group (Figure 1A). These findings were mirrored in fully mature neurons (day 14), where YGD alone significantly increased viability starting at 10 μg/mL and continuing up to 50 μg/mL. Although no significant differences were observed at 100 μg/mL, a downward trend in viability was evident, indicating possible toxicity. Under H_2_O_2_ conditions, YGD at 10, 25, and 50 μg/mL significantly enhanced cell viability (Figure 1B). Based on these results, the optimal concentration range for the neuroprotective effects of YGD was 10–50 μg/mL. To further validate these findings, live/dead assays were performed on days 3 (Figure 1C) and 14 (Supplementary Figure S1). On day 3, quantitative analysis revealed a significant increase in dead cell intensity in the H_2_O_2_ group compared with the blank group, while YGD treatment, in contrast to H_2_O_2_ treatment, led to a dose-dependent reduction in dead cells. The live/dead ratio also showed a significant improvement with YGD at 25 and 50 μg/mL, compared with the H_2_O_2_ treatment. On day 14, qualitative analysis from representative images confirmed these trends, although accurate quantification was challenging due to the extensive neurite outgrowth and network formation typical of mature neurons. Consequently, only representative images from the day-14 live/dead assay are provided in Figure S1.

### 3.2. YGD Enhances Neurite Outgrowth in H_2_O_2_-Stressed Hippocampal Neurons

**Figure 2:**
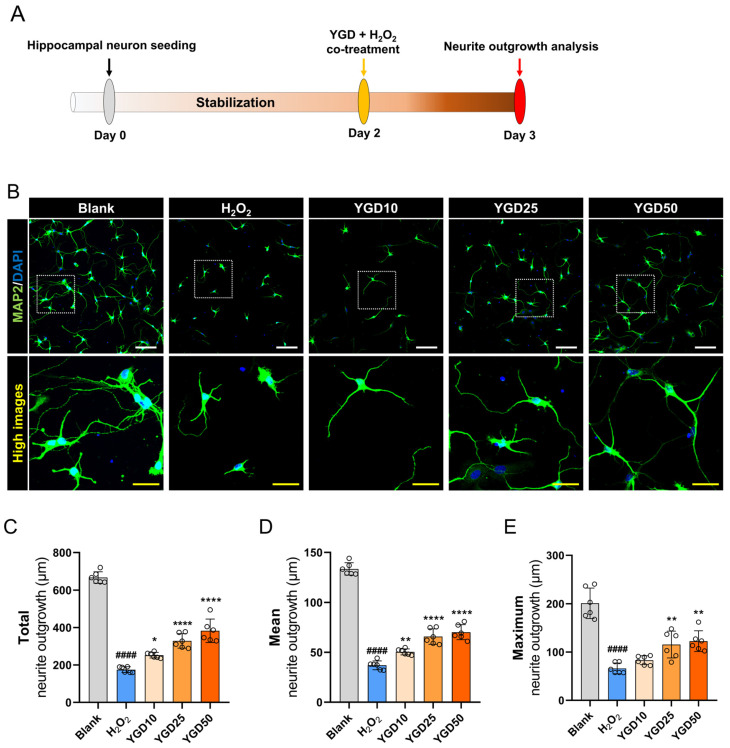
YGD enhances neurite outgrowth in H_2_O_2_-stressed hippocampal neurons. (**A**) Schematic representation of the experimental setup: hippocampal neurons were cultured for 1 day and treated with optimal YGD concentrations (10, 25, and 50 μg/mL) in the presence of H_2_O_2_. After 24 h, neurite outgrowth was analyzed using immunocytochemistry with MAP2 antibody. (**B**) Representative immunocytochemical images showing neurite outgrowth in MAP2-positive cells across the different treatment groups. The white scale bar represents 100 μm, and the yellow scale bar represents 35 μm. (**C**–**E**) Quantitative analysis of total, mean, and maximum neurite lengths across the different treatment groups. Data are presented as the mean ± standard deviation. Significant variations were evaluated using one-way ANOVA coupled with Tukey’s post hoc analysis. Significance levels are denoted as follows: #### *p* < 0.0001 compared with the blank group; * *p* < 0.05, ** *p* < 0.01, and **** *p* < 0.0001 compared with the H_2_O_2_ group.

Having identified the optimal YGD concentrations (10, 25, and 50 μg/mL), we next evaluated the effects of YGD on neurite outgrowth in the 3-day cultured hippocampal neurons (Figure 2A). Immunocytochemistry with MAP2 antibodies showed that H_2_O_2_-treated neurons exhibited significantly shorter neurites in MAP2-positive cells than those in non-treated neurons. In contrast, YGD co-treatment promoted a dose-dependent increase in neurite length (Figure 2B). Quantitative analysis confirmed that H_2_O_2_ significantly reduced neurite outgrowth, while YGD at 25 and 50 μg/mL significantly increased total, mean, and maximum neurite lengths. Although the 10 μg/mL dose showed a positive trend, it did not reach statistical significance (Figure 2C–E).

### 3.3. YGD Enhances Synaptic Integrity via Increased PSD-95 and Synapsin-1 Expressions

**Figure 3:**
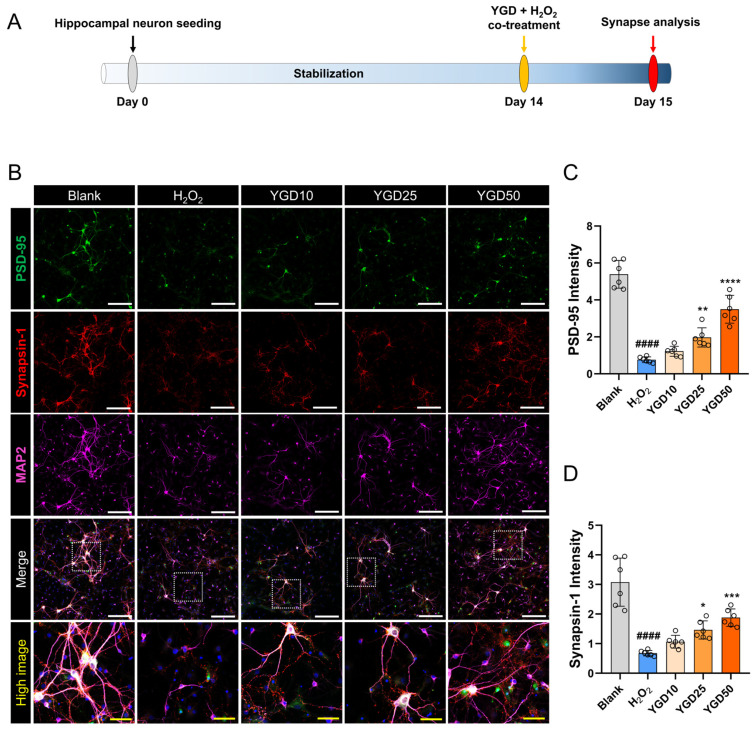
YGD enhances synaptic integrity by increasing PSD-95 and synapsin-1 expressions under H_2_O_2_-induced oxidative stress. (**A**) Schematic representation of the experimental setup: hippocampal neurons were cultured for 14 days and then treated with YGD at concentrations of 10, 25, and 50 μg/mL in the presence of H_2_O_2_ for 24 h. Following treatment, the cells were stained for PSD-95 (a postsynaptic marker), synapsin-1 (a presynaptic marker), and MAP2 (a neuronal marker). (**B**) Representative immunocytochemical images showing the expressions of PSD-95 and synapsin-1 in MAP2-positive neurons across different treatment groups. The white scale bar represents 200 μm, and the yellow scale bar represents 50 μm. (**C**,**D**) Quantitative analysis of PSD-95 and synapsin-1 expression based on the immunocytochemical images. Data are presented as the mean ± standard deviation. Significant variations were evaluated using one-way ANOVA coupled with Tukey’s post hoc analysis. Significance levels are denoted as follows: #### *p* < 0.0001 compared with the blank group; * *p* < 0.05, ** *p* < 0.01, *** *p* < 0.001, and **** *p* < 0.0001 compared with the H_2_O_2_ group.

To investigate the impact of YGD on synapse formation under H_2_O_2_-induced oxidative stress, hippocampal neurons cultured for 14 days were stained for PSD-95 (a postsynaptic marker), synapsin-1 (a presynaptic marker), and MAP2 (a neuronal marker) (Figure 3A). The representative images illustrate that H_2_O_2_ treatment led to a marked reduction in the expressions of synaptic markers PSD-95 and synapsin-1, accompanied by damage to MAP2-positive neurons. In contrast, YGD treatment showed a dose-dependent and substantial increase in the expression of PSD-95 and synapsin-1, alongside enhanced MAP2 expression (Figure 3B). Quantitative analysis revealed that H_2_O_2_ treatment significantly reduced the expression of PSD-95 and synapsin-1, indicative of disrupted synaptic integrity. However, compared with H_2_O_2_ treatment, YGD treatment resulted in a dose-dependent increase in the expression of these synaptic markers, with significant effects at 25 and 50 μg/mL. Although the 10 μg/mL dose showed an upward trend, it did not reach statistical significance (Figure 3C,D).

### 3.4. YGD Enhances Antioxidant Defense via Nrf2 Upregulation and Modulates Stress Response by Reducing Phosphorylated ERK Levels

**Figure 4:**
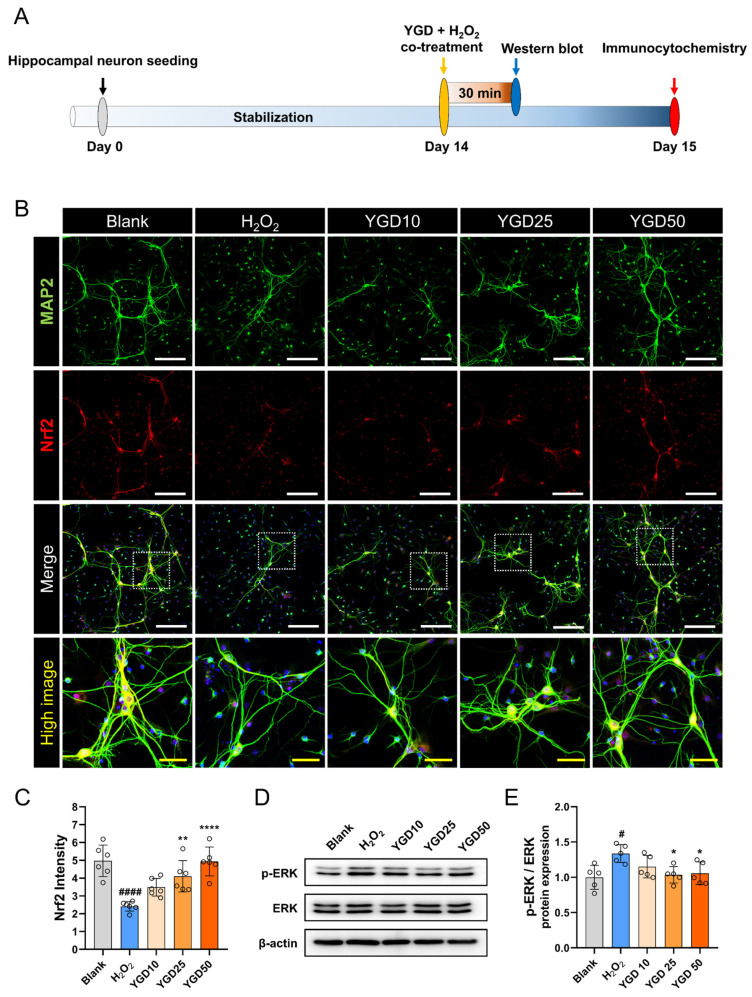
YGD enhances antioxidant defense via Nrf2 upregulation and modulates the stress response by reducing p-ERK levels under H_2_O_2_-induced oxidative stress. (**A**) Schematic representation of the experimental setup: To assess the effects of YGD on Nrf2 and ERK protein expression in hippocampal neurons under H_2_O_2_-induced oxidative stress, two distinct timelines were followed, with drug treatment performed either 30 min or 24 h prior to sampling for Western blot or immunocytochemistry analysis. (**B**,**C**) Representative immunocytochemical images and quantitative analysis of Nrf2 expression across different groups. The white scale bar represents 200 μm, and the yellow scale bar represents 50 μm. (**D**,**E**) Representative Western blot bands showing the expression of p-ERK and total ERK across different groups, along with quantitative analysis of the p-ERK/ERK ratio. Data are presented as the mean ± standard deviation. Significant variations were evaluated using one-way ANOVA coupled with Tukey’s post hoc analysis. Significance levels are denoted as follows: # *p* < 0.05 and #### *p* < 0.0001 compared with the blank group; * *p* < 0.05, ** *p* < 0.01, and **** *p* < 0.0001 compared with the H_2_O_2_ group.

To investigate the protective effects of YGD against oxidative stress, we assessed the expression of Nrf2 and ERK proteins using immunocytochemistry and Western blotting, following two distinct experimental timelines (Figure 4A). Nrf2 is a crucial regulator of the antioxidant defense system [32], while ERK is vital in stress response and cell survival signaling pathways [33]. Notably, increased phosphorylated ERK (p-ERK) is closely associated with neurodegenerative processes, including AD progression [34]. Nrf2 expression was significantly reduced in H_2_O_2_-treated cells compared with the blank cells, indicating an impaired antioxidant response. However, YGD treatment dose-dependently restored Nrf2 expression, with significant increases observed at concentrations of 25 and 50 μg/mL in contrast to the H_2_O_2_ group (Figure 4B,C). Moreover, Western blot analysis revealed that H_2_O_2_ treatment caused a marked increase in p-ERK expression, indicating an elevated stress response. YGD co-treatment, however, led to a dose-dependent reduction in p-ERK levels, with significant decreases observed at 25 and 50 μg/mL (Figure 4D,E). This highlights its potential role in protecting cells from oxidative damage.

### 3.5. YGD Attenuates AD-Related Pathological Features by Modulating p-GSK3β, p-tau, and Aβ Expression in H_2_O_2_-Stressed Hippocampal Neurons

**Figure 5:**
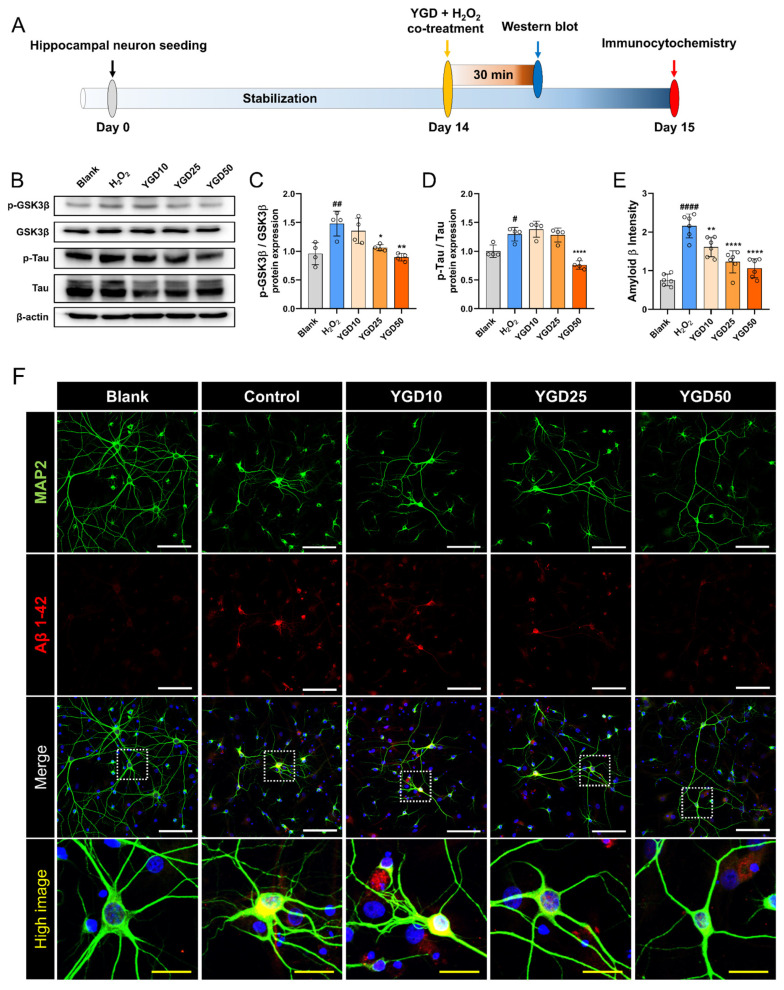
YGD attenuates Alzheimer’s disease-related pathological features by modulating p-GSK3β, p-tau, and Aβ expression in H_2_O_2_-stressed hippocampal neurons. (**A**) Schematic representation of the experimental setup: hippocampal neurons were treated with H_2_O_2_ to induce oxidative stress and co-treated with YGD at concentrations of 10, 25, and 50 μg/mL. Expression levels were evaluated on the basis of two distinct experimental timelines. (**B**) Representative Western blot band images showing the expression of p-GSK3β, total GSK3β, p-tau, and total tau across different treatment groups. (**C**,**D**) Quantitative analysis of Western blot results, depicting the p-GSK3β/GSK3β ratio and the p-tau/tau ratio across treatment groups. (**E**,**F**) Quantitative analysis of Aβ 1–42 expression intensity and representative immunocytochemical images of Aβ 1–42 expression using MAP2 and Aβ antibodies. The white scale bar represents 100 μm, and the yellow scale bar represents 25 μm. Significance levels are denoted as follows: # *p* < 0.05, ## *p* < 0.01 and #### *p* < 0.0001 compared with the blank group; * *p* < 0.05, ** *p* < 0.01, and **** *p* < 0.0001 compared with the H_2_O_2_ group.

To further investigate the effects of YGD on AD-related pathological mechanisms, we examined its impact on the expressions of GSK3β, p-tau, and Aβ in H_2_O_2_-treated hippocampal neurons using two distinct experimental timelines (Figure 5A). H_2_O_2_ treatment activated GSK3β in hippocampal neurons, as indicated by the increased p-GSK3β levels, whereas a significant decrease was observed with YGD treatment at 50 μg/mL (Figure 5B,C). Similarly, H_2_O_2_ treatment increased p-tau expression, a key marker of NFT formation, which is a hallmark of AD. YGD co-treatment significantly reduced p-tau levels, particularly at 50 μg/mL (Figure 5B,D). Additionally, immunocytochemical analysis using MAP2 and Aβ 1–42 antibodies showed a significant increase in Aβ expression in H_2_O_2_-treated neurons, consistent with the neurotoxic effects of oxidative stress observed in AD. YGD treatment reduced Aβ levels in a dose-dependent manner, with significant reductions at all tested concentrations (Figure 5E,F).

### 3.6. Molecular Docking Analysis of YGD-Derived Compounds Targeting GSK3β

**Figure 6:**
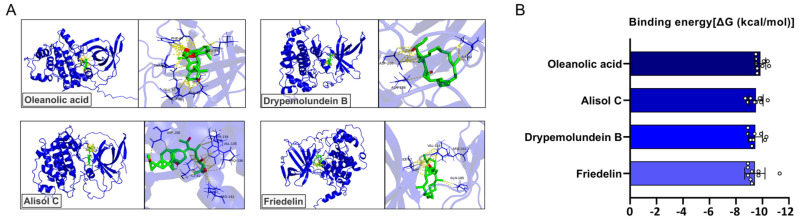
Molecular docking analysis of YGD-derived phytochemicals targeting GSK3β. (**A**) Representative 3D docking poses of the four top-ranked phytochemicals from each of the ten medicinal herbs comprising YGD within the ATP-binding site of GSK3β. (**B**) Binding affinity values (kcal/mol) of ten docking poses for the four top-ranked phytochemicals from each of the ten medicinal herbs comprising YGD within the ATP-binding site of GSK3β. Detailed docking poses of the remaining YGD-derived compounds, excluding those shown in the main figure are provided in the Supplementary Materials.

To elucidate the molecular basis of the effects of YGD on AD pathology, we conducted molecular docking studies targeting GSK3β, a key kinase involved in tau phosphorylation. Based on the previous literature, major phytochemicals from each of the ten medicinal herbs comprising YGD were selected, and molecular docking analyses were performed targeting GSK3β (Supplementary Files). For each herb, the compound with the highest binding affinity to GSK3β was identified (Figure 6A). Oleanolic acid derived from *Cornus officinalis* exhibited the strongest binding affinity among all tested compounds, with a binding energy of −9.86 ± 0.40 kcal/mol. It formed stable interactions with key GSK3β residues, including ARG96, ASN95, GLU97, and LEU88, suggesting a strong and specific interaction within the active pocket (Figure 6B and Table 2). Several other compounds also demonstrated high binding affinities to GSK3β: Alisol C from Alisma canaliculatum (−9.51 ± 0.55 kcal/mol), Drypemolundein B from Cervi pantotrichum (−9.47 ± 0.54 kcal/mol), and Friedelin from Wolfiporia extensa (−9.43 ± 0.77 kcal/mol). These ligands engaged key catalytic residues, such as ASP200, ILE62, and ARG141, indicating potential inhibitory activity. Additional compounds, such as Campestanol (*Dioscorea polystachya*, −9.23 ± 0.48 kcal/mol), Stigmasterol (*Angelicae radix*, −9.17 ± 0.31 kcal/mol), and 3-epi-Karounidiol (*Aquilaria agallocha*, −9.13 ± 0.60 kcal/mol), also exhibited favorable binding profiles, reinforcing the possibility of multi-ligand engagement with GSK3β. In contrast, Staphidine (*Rehmannia glutinosa*, −8.02 ± 1.22 kcal/mol) and β-sitosterol (*Moutan cortex*, −7.98 ± 0.36 kcal/mol) showed comparatively lower binding affinities (Figure S2). Nevertheless, both compounds interacted with residues such as GLN185, LYS183, and VAL135, suggesting they synergistically contribute to polypharmacology. These findings indicate that multiple phytochemicals present in YGD are capable of interacting with neurodegeneration-associated targets in the docking analysis. To validate the docking protocol, 6-bromoindirubin-3′-oxime was redocked into GSK3β. The predicted binding affinity ranged from −8.62 to −7.93 kcal/mol, which was stronger than the reported binding affinity of ATP to GSK3β (−6.92 kcal/mol). Structural alignment between the docked pose and the experimentally determined crystal structure (PDB ID: 1UV5) yielded an RMSD value of 0.812 Å (Figure S3).

## 4. Discussion

Oxidative stress plays a crucial role in the progression of AD, closely linked to hallmark pathological features, such as Aβ plaque accumulation, tau hyperphosphorylation, synaptic dysfunction, and neuronal death [11]. In this study, YGD exerted significant neuroprotective effects on hippocampal neurons exposed to H_2_O_2_-induced oxidative stress. These protective effects were evidenced by enhanced cell survival, increased neurite outgrowth, and preservation of synaptic integrity—key indicators of AD-related pathology. Notably, these effects were achieved through a three-tier experimental approach: short-term analysis of neurite outgrowth after 2 days of culturing and co-treatment, a 14-day culture to assess synaptic protein expression, and a rapid 30 min evaluation of AD-related phosphorylated proteins. This timeline can accurately measure the impact of YGD on early neurite formation, mature synapse preservation, and acute stress signaling, and highlights its therapeutic potential in AD [15,16,35,36].

Oxidative stress reduces the expression of key synaptic proteins, such as PSD-95 and synapsin-1, which are critical for synapse formation and plasticity [37]. This downregulation impairs synaptic communication and is closely associated with cognitive decline in AD. In our study, H_2_O_2_ treatment significantly decreased PSD-95 and synapsin-1 levels, indicating synaptic damage. YGD treatment reversed this effect by restoring the expression of these proteins, thereby preserving synaptic integrity under oxidative stress conditions. One of the key findings of this study was the dose-dependent regulation of p-ERK, p-GSK3β, and p-tau by YGD. The increase in p-ERK following H_2_O_2_ exposure is particularly significant, as ERK is a critical component of the MAPK signaling pathway, which regulates cell survival, differentiation, and stress responses [38,39]. Under oxidative stress conditions, excessive ERK activation can contribute to synaptic dysfunction and neuronal death, central features of AD pathology [38]. Our results indicate that YGD, especially at concentrations of 25 and 50 μg/mL, effectively reduced p-ERK levels, suggesting that YGD alleviates ERK-driven neurotoxicity and maintains cellular homeostasis in stressed neurons.

Previous studies have demonstrated that oxidative stress can abnormally activate ERK, which may indirectly contribute to the activation of GSK-3β, promoting tau hyperphosphorylation and Aβ plaque formation, ultimately exacerbating neurodegeneration [40,41]. GSK-3β is a key enzyme involved in tau phosphorylation, and its overactivation leads to the formation of NFTs, a primary pathological hallmark of AD [42,43]. In our study, H_2_O_2_ treatment significantly increased p-GSK3β and p-tau levels, indicating the detrimental effects of oxidative stress on neuronal function. YGD co-treatment mitigated these increases in a dose-dependent manner, with the most pronounced effects observed at 50 μg/mL. Therefore, the ability of YGD to modulate ERK activity may help prevent GSK-3β overactivation, protecting neurons from tau hyperphosphorylation and NFT formation. Additionally, to identify potential active constituents underlying these protective effects, we performed molecular docking analyses on phytochemicals derived from each of the ten medicinal herbs comprising YGD, focusing on their interactions with GSK3β. Among the YGD-derived phytochemicals evaluated, oleanolic acid, a triterpenoid compound derived from *Cornus officinalis*, demonstrated the highest binding affinity to GSK3β (−9.86 ± 0.40 kcal/mol) and formed stable interactions with key residues (ARG96, ASN95, GLU97, and LEU88) within the ATP-binding site. These results suggest that oleanolic acid may contribute to the YGD-mediated attenuation of GSK3β signaling and tau phosphorylation. Consistent with this interpretation, previous studies have reported that oleanolic acid exerts neuroprotective effects in experimental models of AD, including antioxidant and anti-inflammatory actions and modulation of stress-responsive pathways [44,45,46,47]. Importantly, experimental validation of the predicted molecular interactions was not performed in the present study; therefore, the molecular docking results should be interpreted as predictive rather than confirmatory. However, because experimental validation was not performed, the docking results should be interpreted as predictive rather than confirmatory, and further in vitro and in vivo studies—including enzymatic or cell-based functional assays—will be required to determine whether YGD-derived compounds directly inhibit GSK3β activity and to clarify their molecular mechanisms. Additionally, direct comparative experiments with the parent formulations were not performed; the mechanistic features of YGD may be conceptually understood based on the complementary properties previously reported for GJD and YMJ. Collectively, our docking data support the possibility that YGD may act through multi-component, multi-target interactions, with oleanolic acid as one plausible contributor to GSK3β-related effects. Consistent with this interpretation, the concurrent modulation of GSK3β and tau phosphorylation observed in this study supports the hypothesis that YGD may exert an integrated mechanistic effect on AD-related signaling pathways, although the long-term consequences of this modulation remain to be determined.

Aβ plaques are another major pathological feature of AD. Oxidative stress activates β-secretase, an enzyme that cleaves amyloid precursor protein to produce Aβ. Increased oxidative stress accelerates the amyloidogenic pathway, resulting in greater Aβ production [42]. Our immunocytochemical analysis of Aβ 1–42 further supports the potential of YGD to mitigate AD-like features induced by H_2_O_2_. Consistent with oxidative stress, H_2_O_2_ treatment led to significant Aβ accumulation, likely due to enhanced amyloidogenic processing of amyloid precursor protein. YGD co-treatment significantly reduced Aβ levels in a dose-dependent manner, particularly at 25 and 50 μg/mL.

These findings suggest that YGD either interferes with the oxidative stress-induced Aβ production or promotes Aβ clearance, protecting neurons from Aβ-induced toxicity. The ability of YGD to modulate tau and Aβ pathology underscores its potential as a multi-target therapeutic approach for AD. Despite these promising findings, our study has some limitations. While we demonstrated that YGD exerted neuroprotective effects in hippocampal neurons under AD-like pathology, whether these findings can be replicated in vivo remains to be determined. Future studies using AD animal models are warranted to explore the complex interactions between oxidative stress, amyloid deposition, and tau pathology. Additionally, while we showed that YGD enhanced cell survival, promoted neurite outgrowth, and preserved synaptic integrity by regulating p-ERK, p-GSK3β, and p-tau expression, these techniques alone are not sufficient to fully elucidate the precise molecular mechanisms involved. Further studies using pathway-specific inhibitors or genetic manipulation will provide deeper insights into the signaling pathways modulated by YGD. Lastly, the extensive neurite outgrowth observed in fully mature neurons on day 14 highlighted the limitations of the live/dead assay, which may not capture the full scope of neuroprotection in later stages of neuronal development. Future studies could incorporate advanced imaging techniques, such as live imaging for real-time tracking of fluorescently tagged neurites or 3D synaptic modeling, to provide more accurate assessments of neuronal health and connectivity.

## 5. Conclusions

YGD exhibits significant neuroprotective effects in H_2_O_2_-stressed hippocampal neurons, enhancing cell viability, promoting neurite outgrowth, and preserving synaptic integrity. Its ability to modulate key markers of AD pathology—such as p-ERK, p-GSK3β, p-tau, and Aβ—supports its relevance in attenuating oxidative stress-induced neuronal alterations at the cellular level. Nevertheless, further research remains essential to elucidate the precise molecular mechanisms underlying these effects and to validate the therapeutic potential of YGD in *in vivo* models.

## Figures and Tables

**Table 1:** Primary antibodies used for Western blot analysis.

Primary Antibody	Source	Vendor	Dilution Factor
GSK3β	Mouse monoclonal	CST	1:1000
p-GSK3β	Rabbit polyclonal	CST	1:1000
ERK	Rabbit polyclonal	CST	1:1000
p-ERK	Rabbit monoclonal	CST	1:1000
Tau	Guinea pig polyclonal	Synaptic Systems	1:1000
p-Tau	Mouse monoclonal	Invitrogen	1:500
β-actin	Mouse monoclonal	Santa Cruz	1:2000

**Table 2:** In silico docking results of YGD compounds against GSK3β protein.

Herb	Ligand	Binding Energy (kcal/mol)	Ki (µM)	pKi	RMSD (Å)	Amino Acid Residue	Hydrogen Bond Count
*Cornus officinalis*	Oleanolic acid	−9.86 ± 0.40	0.07 ± 0.04	7.22 ± 0.30	6.32 ± 5.56	ARG96 ASN95 GLU97 LEU88 PHE67 THR-8	5.89 ± 1.54
*Alisma canaliculatum*	Alisol C	−9.51 ± 0.55	0.15 ± 0.13	6.97 ± 0.40	5.09 ± 2.62	ARG141 ASP200 ILE62 PRO136 TYR134 VAL135	4.89 ± 2.03
*Cervi pantotrichum*	*Drypemolundein B*	−9.47 ± 0.54	0.15 ± 0.10	6.94 ± 0.39	4.91 ± 2.35	ASN186 ASP200 ILE62	2.89 ± 1.45
*Wolfiporia extensa*	*Friedelin*	−9.43 ± 0.77	0.18 ± 0.11	6.92 ± 0.56	5.24 ± 2.47	ARG141 GLN185 ILE-62 VAL135	2.89 ± 0.93
*Dioscorea polystachya*	Campestanol	−9.23 ± 0.48	0.22 ± 0.15	6.77 ± 0.36	5.34 ± 3.39	ARG141 ASP200 GLY68 ILE62 PRO136 TYR134 VAL135	4.78 ± 1.20
*Angelicae radix*	Stigmasterol	−9.17 ± 0.31	0.21 ± 0.10	6.72 ± 0.23	6.60 ± 2.91	ARG141 ASP200 GLY68 ILE62 PRO136 TYR134 VAL135	5.00 ± 2.12
*Aquilaria agallocha*	3-epi-Karounidiol	−9.13 ± 0.60	0.27 ± 0.15	6.70 ± 0.44	6.25 ± 5.10	ASP200 GLN185 ILE62 LYS85	4.44 ± 1.13
*Musk*	Cholesta-3,5-diene	−8.54 ± 0.33	0.62 ± 0.34	6.26 ± 0.24	5.49 ± 2.23	ASP200 GLN185 ILE62	3.11 ± 1.54
*Rehmannia glutinosa*	Staphidine	−8.02 ± 1.22	3.87 ± 3.86	5.88 ± 0.90	8.37 ± 4.67	ASP200 GLN185 LYS183 SER66	4.67 ± 2.18
*Moutan cortex*	β-sitosterol	−7.98 ± 0.36	1.68 ± 1.10	5.85 ± 0.26	5.04 ± 3.71	ASN64 LYS183 VAL135	3.89 ± 1.76

RMSD—root-mean-square deviation.

## Data Availability

Data are contained within the article and Supplementary Materials.

## Supplementary Materials

The following supporting information can be downloaded at: https://www.mdpi.com/article/10.3390/biology15030294/s1. Figure S1. Effects of YGD on long-term–cultured hippocampal neuron viability assessed by Live/Dead assay; Figure S2. Molecular docking analysis of additional YGD-derived phytochemicals targeting GSK3β; Figure S3. Validation of the docking protocol by redocking 6-bromoindirubin-3′-oxime into GSK3β.

## Abbreviations

The following abbreviations are used in this manuscript:

Aβ	amyloid-β
AD	Alzheimer’s disease
APP	amyloid precursor protein
CCK8	cell counting kit-8
DPBS	Dulbecco’s phosphate-buffered saline
ERK	extracellular signal-regulated kinase
GJD	Gongjin-dan
GSK3β	glycogen synthase kinase
H_2_O_2_	hydrogen peroxide
MAP2	microtubule-associated protein 2
NFT	neurofibrillary tangle
Nrf2	nuclear factor-erythroid 2-related factor-2
PBS	phosphate-buffered saline
ROS	reactive oxygen species
YGD	Yookgong-dan
YMJ	Yukmijihwang
