# Supplementary material for: Neuroprotective Effects of Herbal Formula Yookgong-Dan on Oxidative Stress-Induced Tau Hyperphosphorylation in Rat Primary Hippocampal Neurons
Source: Biology (Basel). 2026 Feb 6;15(3):294. doi: 10.3390/biology15030294 (PMC12896959; doi:10.3390/biology15030294)
Supplement: Supplementary file 1 [file biology-15-00294-s001.zip › Supplementary material.docx]

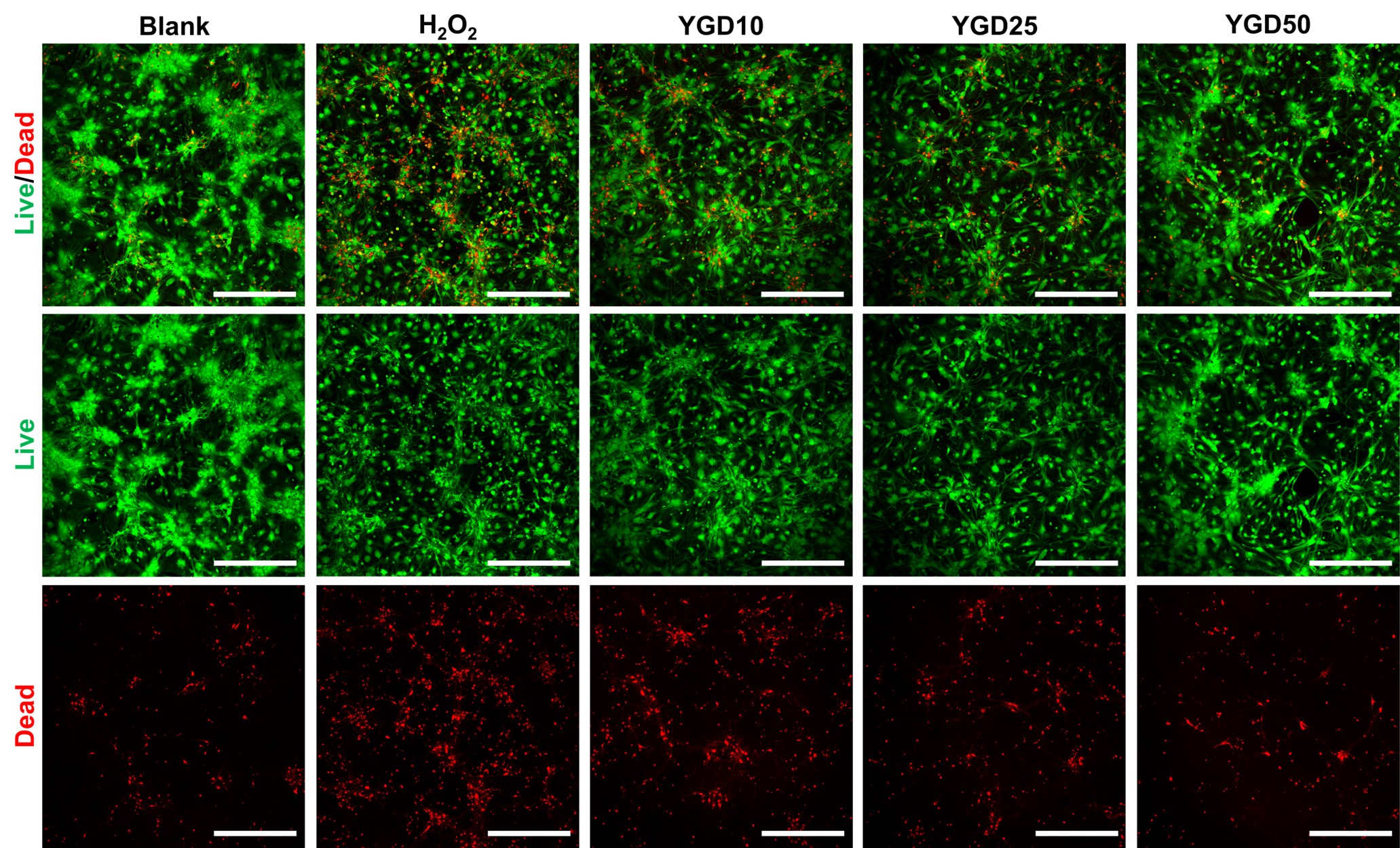


**Figure S1. Effects of YGD on long-term–cultured hippocampal neuron viability assessed by Live/Dead assay.**

Representative images of Live/Dead assay performed on day 15 after 14 d of hippocampal neuron culture, followed by 24 h of treatment with H₂O₂ and three different concentrations of YGD (10, 25, and 50 μg/mL).





**Figure S2.** Molecular docking analysis of additional YGD-derived phytochemicals targeting GSK3β.

(A) Representative three-dimensional docking poses of Campestanol, Cholesta-3,5-diene, Stigmasterol, Staphidine, 3-epi-Karounidiol, and β-sitosterol. (B)The corresponding binding affinity values (kcal/mol) obtained from molecular docking analysis.


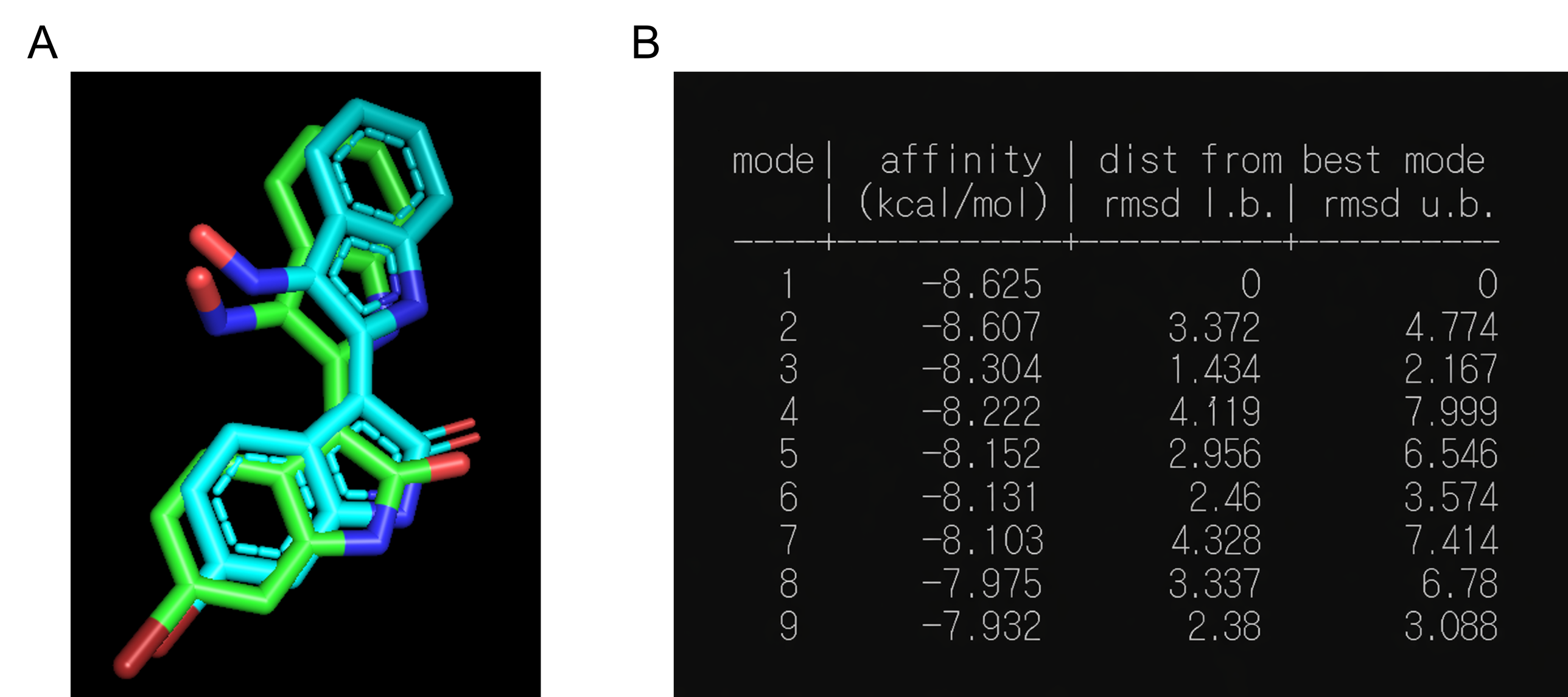


**Figure S3**. Validation of the docking protocol by redocking 6-bromoindirubin-3′-oxime into GSK3β.

(A) The structural alignment between the docked pose (cyan) and the experimentally determined crystal structure of GSK3β in complex with 6-bromoindirubin-3′-oxime (PDB ID: 1UV5; green). (B) The AutoDock Vina output listing binding affinity values and RMSD distances for the generated docking poses. The best-ranked pose exhibited an RMSD value of 0.812 Å relative to the crystal structure.
