# Supplementary material for: Neuroprotective Effects of Herbal Formula Yookgong-Dan on Oxidative Stress-Induced Tau Hyperphosphorylation in Rat Primary Hippocampal Neurons
Source: Biology (Basel). 2026 Feb 6;15(3):294. doi: 10.3390/biology15030294 (PMC12896959; doi:10.3390/biology15030294)
Supplement: Supplementary file 1 [file biology-15-00294-s001.zip › Supplementary Materials.pdf]

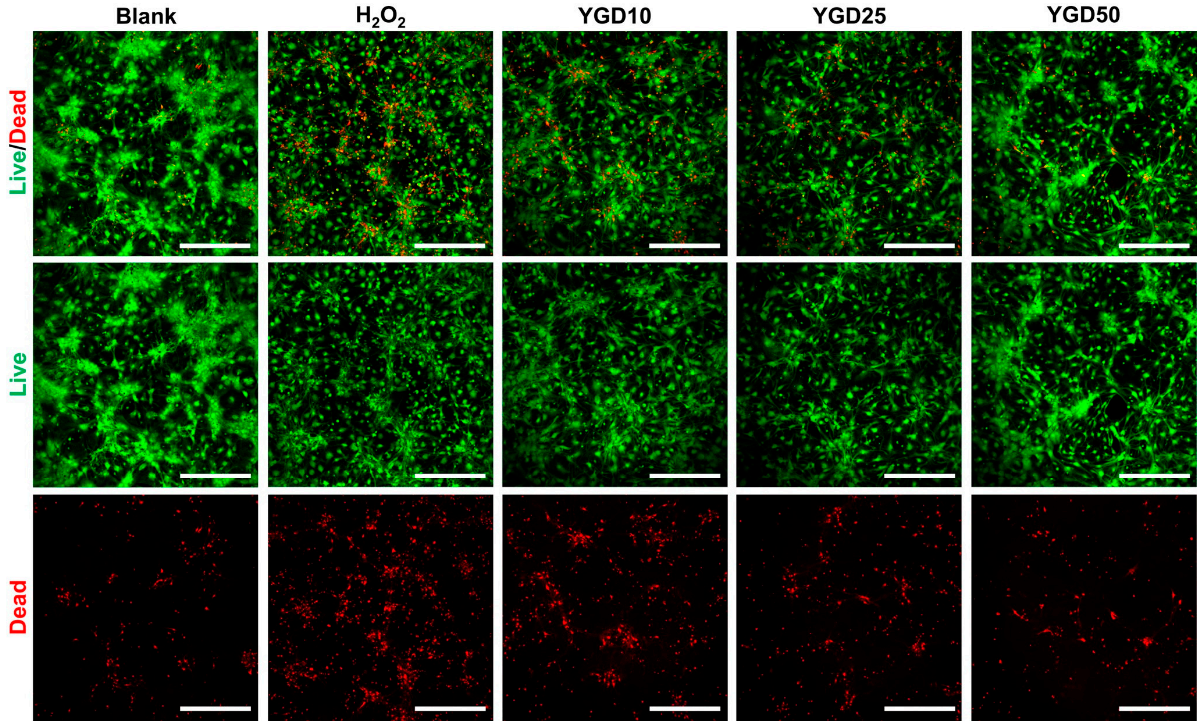

Figure S1. Effects of YGD on long-term–cultured hippocampal neuron viability assessed by Live/Dead assay.

Representative images of Live/Dead assay performed on day 15 after 14 d of hippocampal neuron culture, followed by 24 h of treatment with H_2O_2 and three different concentrations of YGD (10, 25, and 50 μg/mL).

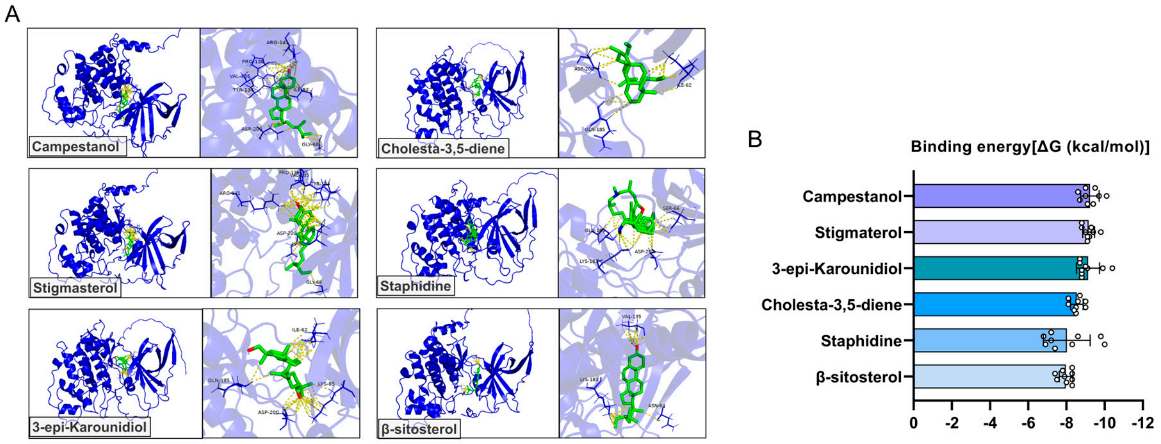

Figure S2. Molecular docking analysis of additional YGD-derived phytochemicals targeting GSK3β.

(A) Representative three-dimensional docking poses of Campestanol, Cholesta-3,5-diene, Stigmasterol, Staphidine, 3-epi-Karounidiol, and β-sitosterol. (B)The corresponding binding affinity values (kcal/mol) obtained from molecular docking analysis.

A

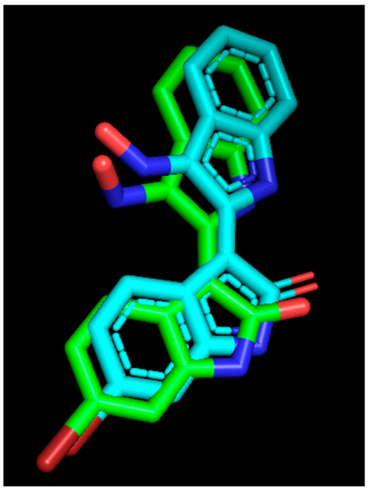

B

mode	affinity (kcal/mol)	dist from best mode rmsd l.b.	rmsd u.b.
1	-8.625	0	0
2	-8.607	3.372	4.774
3	-8.304	1.434	2.167
4	-8.222	4.119	7.999
5	-8.152	2.956	6.546
6	-8.131	2.46	3.574
7	-8.103	4.328	7.414
8	-7.975	3.337	6.78
9	-7.932	2.38	3.088

Figure S3. Validation of the docking protocol by redocking 6-bromoindirubin-3′-oxime into GSK3β.

(A) The structural alignment between the docked pose (cyan) and the experimentally determined crystal structure of GSK3β in complex with 6-bromoindirubin-3′-oxime (PDB ID: 1UV5; green). (B) The AutoDock Vina output listing binding affinity values and RMSD distances for the generated docking poses. The best-ranked pose exhibited an RMSD value of 0.812 Å relative to the crystal structure.
